# Double-Exposure Optical Sectioning Structured Illumination Microscopy Based on Hilbert Transform Reconstruction

**DOI:** 10.1371/journal.pone.0120892

**Published:** 2015-03-23

**Authors:** Xing Zhou, Ming Lei, Dan Dan, Baoli Yao, Jia Qian, Shaohui Yan, Yanlong Yang, Junwei Min, Tong Peng, Tong Ye, Guangde Chen

**Affiliations:** 1 State Key Laboratory of Transient Optics and Photonics, Xi’an Institute of Optics and Precision Mechanics, Chinese Academy of Sciences, Xi’an, 710119, China; 2 School of Science, Xi’an Jiaotong University, Xi’an, 710049, China; 3 Department of Bioengineering, Clemson University, Clemson-MUSC Bioengineering Program, Charleston, South Carolina, 29425, United States of America; Glasgow University, UNITED KINGDOM

## Abstract

Structured illumination microscopy (SIM) with axially optical sectioning capability has found widespread applications in three-dimensional live cell imaging in recent years, since it combines high sensitivity, short image acquisition time, and high spatial resolution. To obtain one sectioned slice, three raw images with a fixed phase-shift, normally 2π/3, are generally required. In this paper, we report a data processing algorithm based on the one-dimensional Hilbert transform, which needs only two raw images with arbitrary phase-shift for each single slice. The proposed algorithm is different from the previous two-dimensional Hilbert spiral transform algorithm in theory. The presented algorithm has the advantages of simpler data processing procedure, faster computation speed and better reconstructed image quality. The validity of the scheme is verified by imaging biological samples in our developed DMD-based LED-illumination SIM system.

## Introduction

Optical sectioning microscopy can produce a clear image of the focal plane deeply within a thick sample. Many different techniques are specifically designed to improve the quality of optical sectioning **[1–3]**. As a wide-field optical microscopy, structured illumination microscopy (SIM) has found widespread applications for investigation of cell structures and for time-series imaging of living specimen due to its optical sectioning capability and high imaging speed **[4–6].**

The basic idea of SIM for optical sectioning is to decode the in-focus information and remove the background of out-of-focus portion. The most commonly used decoding algorithm was proposed by Neil *et al* **[7]**, in which a sinusoidal fringe is projected on the surface of specimen. For each slice, three raw images with a phase-shift of 2π/3 between two adjacent images are acquired. By taking the root mean square (RMS) of the differences of the adjacent images, an optically sectioned image can be reconstructed. Neil’s algorithm provides a simple way to get the three-dimensional (3-D) reconstruction, but also produces some problems at the same time. The main disadvantage of the RMS algorithm is the demanding of precise phase-shift controlling. Small phase-shift errors may result in the residue fringe in the reconstructed slice**[8]**. In addition, although the image acquisition rate of SIM is close to the wide-field microscopy, the actually imaging rate of SIM can only reach to one third of the wide-field microscopy, because three raw images are required to reconstruct one slice.

**Fig 1:**
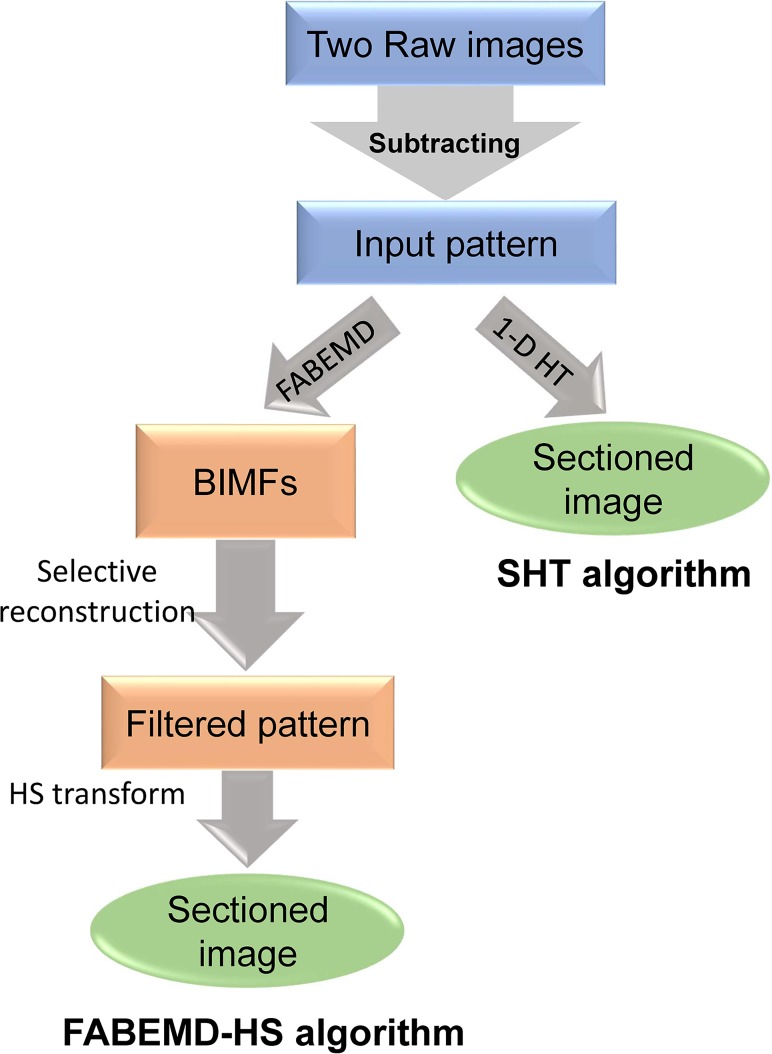
Flowcharts of the FABMED-HS algorithm (left side) and the SHT algorithm (right side).

To circumvent these problems, Santos *et al*. presented a technique called HiLo imaging**[9]**, which uses two widefield images acquired under uniform and structured illumination to synthesize an optically sectioned image. The final optically sectioned image is reconstructed from the fusion of the in-focus high and low frequency image components, which needs special designed high-pass and low pass filters to separate the in focus information from the out-of-focus background to obtain the optimal results. Recently, a new decoding algorithm**[10]** that combines fast and adaptive bi-dimensional empirical mode decomposition (FABEMD)**[11]** method with the Hilbert spiral (HS) transform**[12–13]** was introduced to obtain the sectioned image. This method was originally used in the optical interferometry**[14]** and then in the SIM**[10]** to realize pattern demodulation where a two-dimensional (2-D) HS transform algorithm was adopted. The flowchart of the FABMED-HS algorithm is shown in Fig. 1. In this algorithm, two structured raw images are firstly subtracted to create an input pattern. Then, the input pattern is decomposed into a series of bi-dimensional intrinsic mode functions (BIMFs) and followed by a selective reconstruction procedure, in which some of the BIMFs are filtered out to generate a band-pass filtered pattern, which is used to obtain the sectioned image by the 2-D HS transform eventually. The FABEMD-HS algorithm is well promising and provides a new route to address the optically-sectioned reconstruction problem, especially in processing highly scattering objects with strong signal noise. However, it still remains some limitations such as time consuming**[15]** even some of these issues were considerably improved.**[16]**

In this paper, we propose a one-dimensional (1-D) Sequence Hilbert Transform (SHT) algorithm to decode the in-focus information. Here, only two structured sub-images with arbitrary phase-shift are needed to calculate a single slice, thus the total imaging acquisition time can be reduced by 33% in theory compared to the RMS scheme. While our reconstruction process does not contain the bi-dimensional empirical mode decomposition procedure, the whole reconstruction process is automatic and fast, especially in calculating large-size images. The validity of the SHT algorithm is demonstrated by imaging pollen grains in our DMD-based LED-illumination SIM system.**[17]**

## Methods

A key step of SIM is to project a sinusoidal fringe onto the specimen of interest. Then the captured structured images can be decomposed into the in-focus and the out-of-focus components:

$$I_{cap}(x,y)=I_{n}(x,y)+I_{m}(x,y)\cdot \text{sin}(2\text{π}vx+\varphi ),$$ (1)

where *I*_*n*_ is the out-of-focus background, *I*_*m*_ is the in-focus information, *ν* and *φ* are the spatial frequency and initial phase of the projected sinusoidal fringe, respectively. Because the intensity of the out-of-focus background *I*_*n*_ remains a constant, we can subtract two phase-shifting raw images to eliminate the background *I*_*n*_. Thus, we obtain an input image *I*_*s*_ with sinusoidal amplitude modulation described in Eq. (2):

$$I_{s}(x,y)=I_{m}(x,y)\cdot \text{cos}(\text{2π}vx+\varphi_{s}),$$ (2)

where *φ*_*s*_ is the mean value of the two arbitrary initial phases of the two raw images. The next step is to demodulate the sinusoidal amplitude and obtain the in-focus information *I*_*m*_ from Eq. (2). It is seen that *I*_*m*_ can be solved out by Eq. (2) directly. However, in this way, *I*_*m*_ becomes very sensitive to the precision of *φ*_*s*_. Thus, *I*_*s*_ must be decoded by other ways to eliminate the residue sinusoidal pattern. Here, we utilize the Hilbert transform (HT) and construct a complex analytical signal *I*_*A*_ that is presented in the form of:

$$I_{A}(x,y)=I_{s}(x,y)+i\cdot I_{SH}(x,y),$$ (3)

where *i* is the imaginary unit and the imaginary part *I*_*SH*_ of *I*_*A*_ is the Hilbert transform of the input pattern *I*_*S*_. In optical interferometry, the interferograms may contain complex structures including different frequency components. Therefore, as the description of FABEMD-HS algorithm in Ref.**[14]**, the decoding process must be based on the 2-D Hilbert transform. However, in SIM, the projection fringe contains only single spatial frequency in one orientation (either *x* direction or *y* direction). So, the 2-D image *I*_*S*_ can be simplified as the combination of a sequence of 1-D sinusoidal amplitude modulation signals. In this case, the 1-D signal processing algorithm can be used for the 2-D image demodulation. In 1-D signal analysis, Hilbert transform is a powerful tool to achieve demodulation signal. Based on the characteristic of Hilbert transform, the Hilbert transform of a cosine-modulated function becomes is a sine-modulated function:

$$\begin{array}{l} HT_{x}\{b(x)\cdot \text{cos}\;x\}=\frac{1}{2}[HT_{x}\{b(x)\cdot e^{ix}\}+HT_{x}\{b(x)\cdot e^{-ix}\}]\\ \;=\frac{1}{2}[-i\cdot b(x)e^{ix}+i\cdot b(x)e^{-ix}]\;,\\ \;=b(x)\cdot \text{sin}\;x \end{array}$$ (4)

where *HT*_*x*_{} denotes the Hilbert transform operation in *x* direction. Applying the 1-D Hilbert transform to the *I*_*S*_, we obtain the analytical signal:

$$\begin{array}{l} I_{A}(x,y)=I_{S}(x,y)+i\cdot HT_{x}\{I_{S}(x,y)\}\\ \;=I_{m}(x,y)\;\text{cos}(2\pi vx+\varphi_{s})+i\cdot I_{m}(x,y)\;\text{sin}(2\pi vx+\varphi_{s})\;, \end{array}$$ (5)

Finally, the optically sectioned image *I*_*m*_ can be obtained by:

$$I_{m}(x,y)=\left|I_{A}(x,y)\right|\;.$$ (6)

As shown in Fig. 1, the proposed SHT algorithm avoids the bi-dimensional empirical mode decomposition (BEMD) and band-pass filtration operations in the FABMED-HS algorithm. As a result, the SHT algorithm permits a fast and efficient way to obtain optically-sectioned image, especially in imaging of large-size and thick samples.

## Experimental Setup

**Fig 2:**
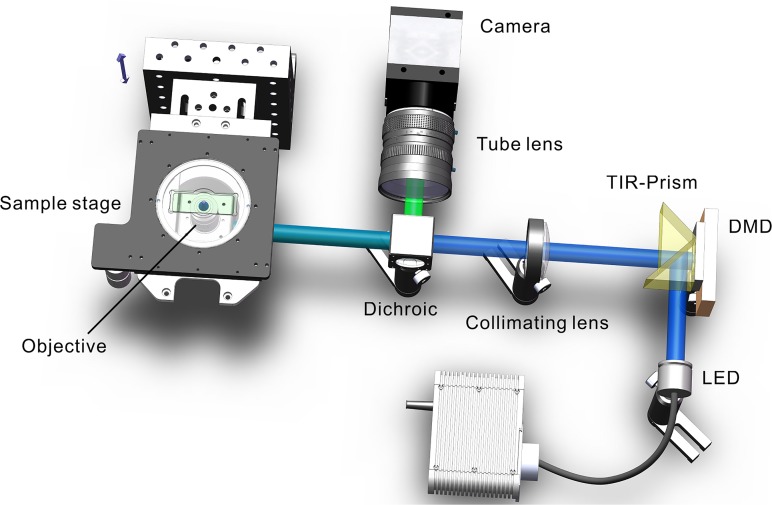
Scheme of the DMD-based LED-illumination optical sectioning SIM system. The binary fringe pattern on DMD is de-magnified and projected onto the specimen through a collimating lens and a microscope objective lens. Higher orders of spatial frequencies of the binary fringe are naturally blocked by the optics, leading to a sinusoidal fringe illumination in the sample plane. Fluorescence light from the specimen is then imaged onto the sCMOS camera.

To demonstrate the feasibility of the above proposed approach, we carried out the following experiment. In our previous study, we had set up a SIM system by using a DMD for fringe projection and a low-coherence LED light for illumination. The experimental setup is illustrated in Fig. 2. A blue LED with central wavelength of 450 nm is employed as the illumination source. The LED light enters the TIR-Prism and illuminates the DMD chip. The reflected light from the DMD chip passes through the TIR-Prism, collimated by an achromatic lens, then passes through a dichroic filter lens (450nm long-pass in reflection) and then focused by a 20× Objective (Plan Fluor, NA0.45, Nikon Inc., Japan) to illuminate the sample. In order to obtain the optimal optical sectioning ability, the illumination pattern loaded on the DMD was set to six pixels per period, and the phase-shift can be adjusted π/3, 2π/3, π, 4π/3 and 5π/3. The sample is mounted on a motorized translation stage (M-405.DG, Physik Instruments Inc., Germany) that can be moved axially in a minimum step of 50 nm. A sCMOS camera with a maximum full-frame rate of 100 fps (Flash4.0, 2048×2048 pixels, 16bits gray-level, Hamamatsu Inc., Japan) is used to capture the fluorescence image. In the traditional RMS scheme, for each axial plane in z-scanning, three exposures for three different phase-shifted patterns are required. Whereas, the proposed SHT approach requires only two exposures, thus the data acquisition time can be reduced by nearly 1/3 compared to the RMS scheme.

## Results

**Fig 3:**
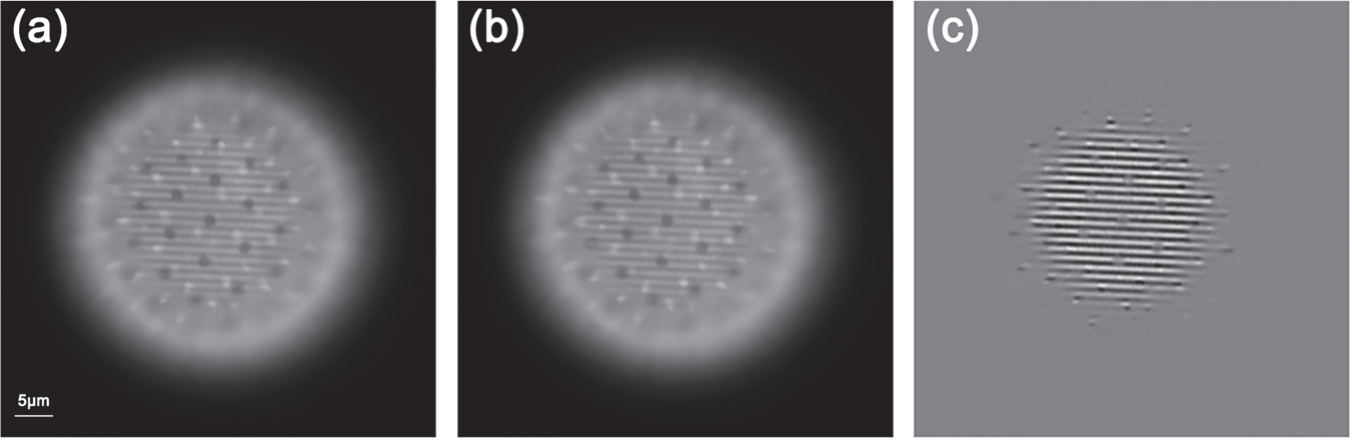
Raw image frames (a) (b) under structured illumination of the pollen grain with phase-shift by 2π/3, and the input image (c) obtained by subtracting (a) and (b). The scale bar is 5μm.

**Fig 4:**
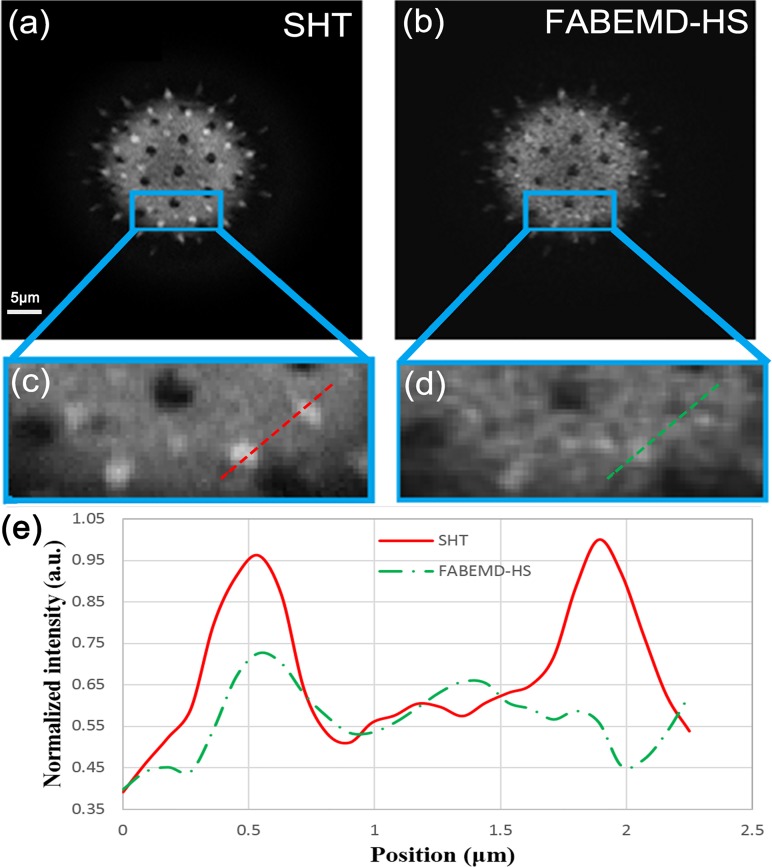
The reconstructed sectioned images of the pollen grain by the proposed SHT algorithm (a), and the FABEMD-HS algorithm (b). Magnifications of the boxed regions of (a) and (b) are shown in (c) and (d), respectively. Intensity profiles along the dashed lines indicated in (c) and (d) are plotted in (e). The scale bar is 5μm.

The tested specimen is a mixed pollen grain specimen purchased from Carolina Biological Supply Company (Burlington, USA), which exhibits strong auto-fluorescence under the excitation of 450 nm LED light. In order to obtain the best signal-to-noise ratio of image **[18]**, all of the raw images were captured by overwriting the 65536 gray scales of the dynamic range of 16bits gray depth of the camera in exposure time of 10 ms. Fig. 3(A) and 3(B) present the captured two raw images of the pollen grain with a phase-shift of 2π/3. As described above, the subtracting operation between Fig. 3(A) and 3(B) generates an input image *I*_*S*_ that contains the desirable in-focus information shown in Fig. 3(C). Using the same input image *I*_*S*_, we reconstruct the sectioned image by using our proposed SHT algorithm (Fig. 4(A)) and the FABEMD-HS algorithm (Fig. 4(B)), respectively. In the FABEMD-HS algorithm, we decompose the *I*_*S*_ into nine BIMFs and selectively reconstruct the optimal band-pass filtered pattern to obtain the sectioned image according to Ref. **[14]**. A subset of the data is enlarged in Fig. 4(C) and 4(D). To make a quantified comparison of reconstruction quality, the intensity profiles along the dashed lines of selected parts in Fig. 4(C) and 4(D) are plotted in Fig. 4(E). It is clear that the SHT algorithm provides higher contrast and more detailed information. By comparing the data processing speed, The FABEMD-HS algorithm takes 15.008s to calculate one slice image (2048×2048 pixels) by using a PC (Intel Core i7-3630QM 2.4GHz processor and 8GB RAM) with the Matlab software (R2013a) under Windows 7 (SP1) x64 operating system, whereas the SHT algorithm takes only 0.151s under the same condition.

**Fig 5:**
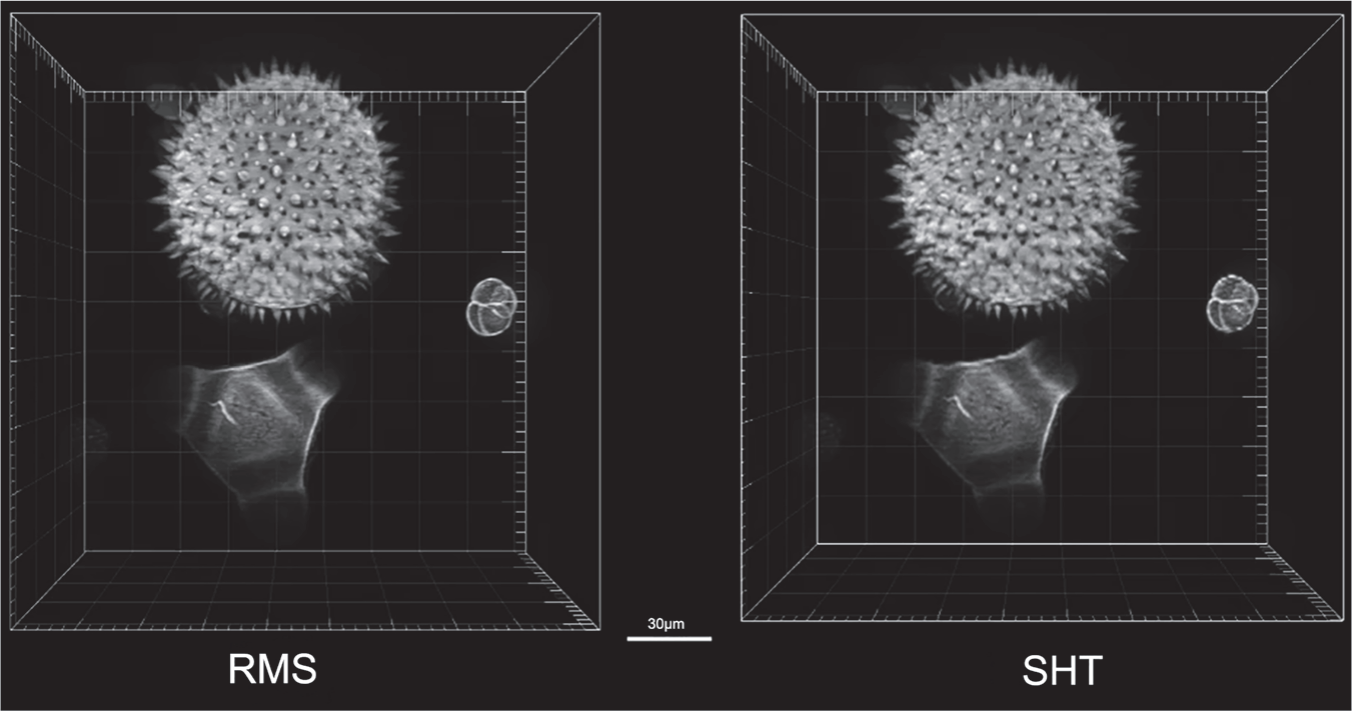
3-D reconstructed images of the mixed pollen grains by using the RMS algorithm (Left) and the SHT algorithm (Right). The scale bar is 30 μm. (S1 Video)

Besides fast speed of data processing, rapid and automated data acquisition of specimens with high quality is the aim of optical sectioning microscopy in practice. Here, we make a comparison on the temporal resolution of the traditional RMS and the proposed SHT approaches. It is noticed that the data processing speed of the SHT algorithm is still slower than the RMS algorithm (SHT: 0.151s per-slice, RMS: 0.044s per-slice). But the temporal resolution of SIM is determined by the data acquisition time rather than the post-processing time, because the reconstruction process always get started after the raw images completely captured. So, in the case of discarding real-time playback of 3D image reconstruction, the SHT approach has its own merit. In the comparing experiment, a sequence of 212 layers (2048×2048pixels/layer) of a volume of 184×184×84.4μm^3^ is sliced with the axial slice interval 400nm. The total data acquisition time for the RMS approach is 8.490s, that is, (10ms exposure time + 0.031ms DMD switching time) × 3 patterns × 212 layers + 10ms Z-stage settling time × 211 axial slice intervals. The SHT approach costs 6.363s, that is, (10ms exposure time + 0.031ms DMD switching time) × 2 patterns × 212 layers + 10ms Z-stage settling time × 211 axial slice intervals. The total image acquisition time of the SHT approach is reduced by 25% compared to the traditional RMS approach. The reason of improvement not reaching 33% is because the speed of data acquisition is limited by the translation stage. At the present version, we used a motorized translation stage with a 10ms settling time and a minimum step of 50nm. Higher acquisition speed can be achieved by using the faster Piezo stage. For example, an existing Piezo positioning system with a 2ms settling time will allow for about 4.675s per volume for 212 slicing layers by using the SHT approach and 6.801s by using the RMS approach, respectively, which corresponds to a 31% improvement. In addition, we present the 3-D images of the mixed pollen grains reconstructed by the RMS and SHT approaches, respectively, as shown in Fig. 5 (S1 Video). The SHT algorithm obtained the nearly same high quality 3-D image as the RMS algorithm.

While short acquisition time and simple operation can be achieved with this type of experiment, its disadvantages should not go unmentioned. First, in a condition of low sampling rate, that is, a period of the fringe is only represented by few of pixels, the discrete Hilbert transform operation will be distorted a lot, in another word, the Eq. (4) will not be strictly satisfied. In that case, the SHT algorithm cannot provide as high resolution as the RMS approach. Second, the post-processing speed of the SHT algorithm is still slower than the RMS algorithm due to the Hilbert transform operation. But this can be significantly improved by using more powerful computer or graphic processing units (GPU) assisted computation. Nevertheless, due to its simplicity and fast imaging capability, the SHT algorithm can still be a useful tool, for example, in fast imaging applications.

## Conclusion

In summary, we have presented a new optical sectioning algorithm in SIM that is based on the 1-D Hilbert transform to realize fast 3-D reconstruction. We experimentally demonstrated the capability of the proposed SHT algorithm by imaging biological samples. We anticipate the proposed SHT algorithm can benefit for high-speed application in observing the 3-D dynamic process of live specimens.

## Supporting Information

S1 Video 3-D reconstructed images of the mixed pollen grains by using the RMS algorithm (Left) and the SHT algorithm (Right). (MOV)
